# Scalable microphysiological system to model three-dimensional blood vessels

**DOI:** 10.1063/1.5090986

**Published:** 2019-06-21

**Authors:** Mees N. S. de Graaf, Amy Cochrane, Francijna E. van den Hil, Wesley Buijsman, Andries D. van der Meer, Albert van den Berg, Christine L. Mummery, Valeria V. Orlova

**Affiliations:** 1 Department of Anatomy and Embryology, Leiden University Medical Center, Einthovenweg 20, 2333 ZC Leiden, The Netherlands; 2 Applied Stem Cell Technologies, University of Twente, P.O. Box 217, 7500 AE Enschede, The Netherlands; 3 BIOS Lab on a Chip, University of Twente, P.O. Box 217, 7500 AE Enschede, The Netherlands

## Abstract

Blood vessel models are increasingly recognized to have value in understanding disease and drug discovery. However, continued improvements are required to more accurately reflect human vessel physiology. Realistic three-dimensional (3D) *in vitro* cultures of human vascular cells inside microfluidic chips, or vessels-on-chips (VoC), could contribute to this since they can recapitulate aspects of the *in vivo* microenvironment by including mechanical stimuli such as shear stress. Here, we used human induced pluripotent stem cells as a source of endothelial cells (hiPSC-ECs), in combination with a technique called viscous finger patterning (VFP) toward this goal. We optimized VFP to create hollow structures in collagen I extracellular-matrix inside microfluidic chips. The lumen formation success rate was over 90% and the resulting cellularized lumens had a consistent diameter over their full length, averaging 336 ± 15 *μ*m. Importantly, hiPSC-ECs cultured in these 3D microphysiological systems formed stable and viable vascular structures within 48 h. Furthermore, this system could support coculture of hiPSC-ECs with primary human brain vascular pericytes, demonstrating their ability to accommodate biologically relevant combinations of multiple vascular cell types. Our protocol for VFP is more robust than previously published methods with respect to success rates and reproducibility of the diameter between- and within channels. This, in combination with the ease of preparation, makes hiPSC-EC based VoC a low-cost platform for future studies in personalized disease modeling.

## INTRODUCTION

Blood vessels are lined with endothelial cells (ECs) and surrounded by mural cells called smooth muscle cells or pericytes. The interaction between mural cells and ECs provides many vessels with stability and abnormal interactions can lead to conditions such as hemorrhage, vascular dementia, and chronic infection.^1–3^ Furthermore, EC and mural cell interaction influences the selectivity of the barrier, which determines whether compounds can enter or are excluded from an organ, and they are therefore important factors in drug efficacy and tissue selectivity.^4,5^ Studying interaction between ECs and mural cells can be complex; for instance, combining three-dimensional (3D) geometry and controlled fluid flow is challenging *in vitro*. Pseudocapillary vascular networks can be generated when culturing vascular cells in conventional two-dimensional (2D) tissue culture plastic dishes but these lack the lumen of vasculature *in vivo.*^6,7^ By contrast, ECs can self-organize in 3D culture environments into complex vascular capillary networks in which a perfusable lumen develops with diameters ranging from approximately 10 *μ*m (the size of an *in vivo* capillary) to 100 *μ*m.^8^ However, precise control of the network geometry during self-organization is challenging and this creates intrinsic variation in wall shear stress when fluid flow is introduced. Wall shear stress is an important determinant of the vascular function.^9^ Its magnitude can be estimated by assuming that blood vessels are straight cylinders with a constant flow rate and viscosity, using the following equation:^8,10^

$$\tau =\frac{32\;\mu \;Q}{\pi \;d^{3}},$$ (1)

where τ: shear stress (Pa), *μ*: viscosity of the fluid (Pa s), Q: flow rate (m^3^/s), and d: diameter (m).

**FIG. 1.**
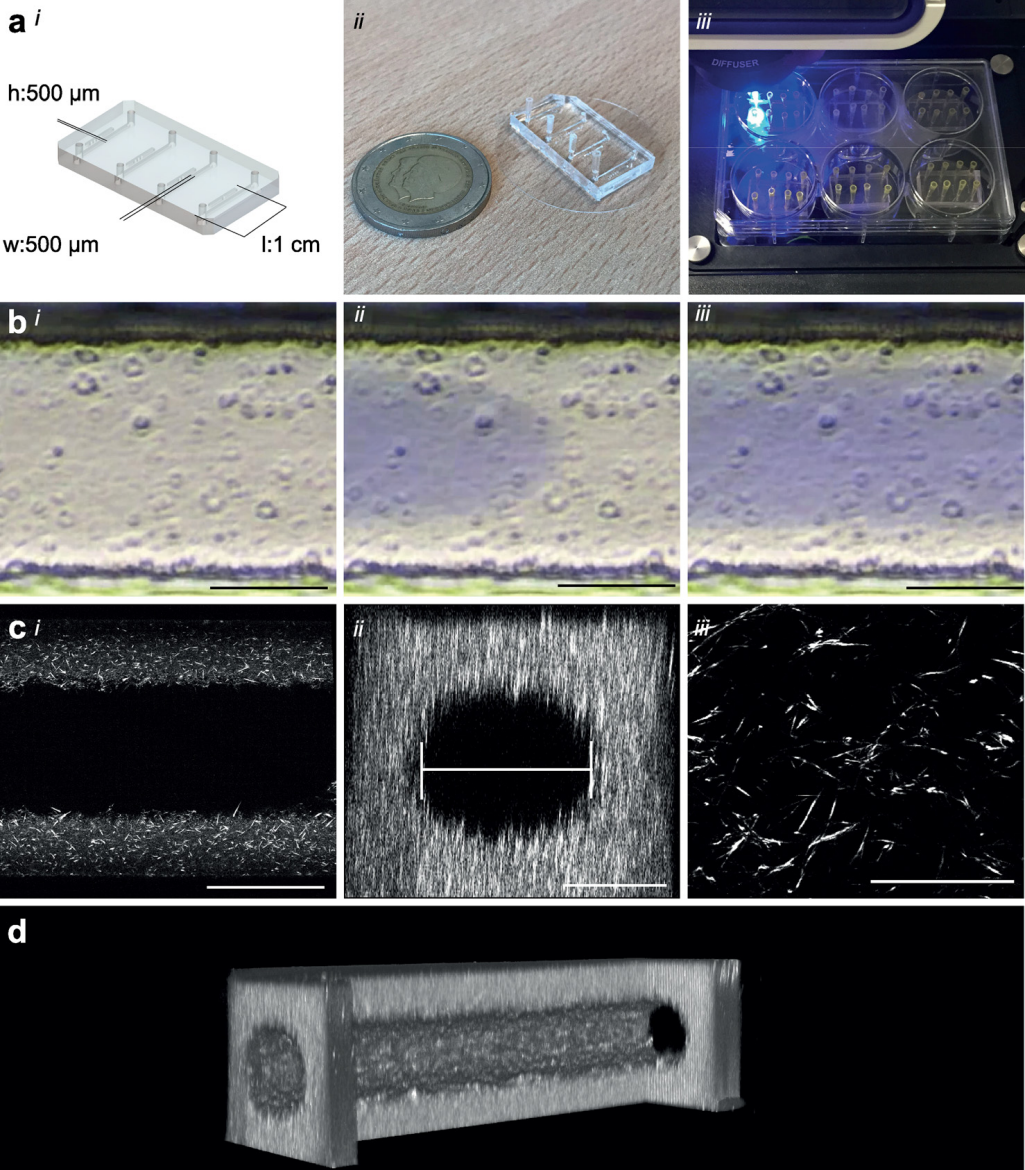
Microfluidic design and patterning collagen scaffold. (a), (i) Schematic of the microfluidic chip showing dimensions and layout of the microfluidic platform, four straight channels on a single chip with designed parameters 500 *μ*m × 500 *μ*m × 1 cm (w × h × l), (ii) Photograph of the real microfluidic device showing four channels ready to be patterned, two-euro coin as size reference, and (iii) Photograph demonstrating ease of use and medium throughput capacity of this setup. The microfluidic device fits in 6-well plates allowing analyses medium throughput in a conventional biological workflow. Manual preparation of patterning of these 24 lumens is typically 10 min. (b) Time-lapse of Viscous finger patterning images showing PBS with blue food dye traveling through collagen solution in a 500 *μ*m wide channel. (i) t = 0 collagen is injected, (ii) PBS finger travels through the channel, and (iii) PBS-finger has completely traversed the channel and displaced the center of the collagen. (c), (i) 20× magnification confocal slice of a patterned lumen with 5 mg/ml collagen I; note the absence of the fibril structure in the center where the “finger” displaced the collagen. (ii) XZ-reconstruction showing the flow field of the scaffold. The diameter is determined by the widest slice of a reconstructed image; (iii) 63× magnification showing a detailed view of the fibril structure of collagen I. (d) 3D cut-out reconstruction of a 2-photon second harmonic generation image showing the collagen scaffold. One side has been cut out to demonstrate the inside of the scaffold. Scale bars, (bi,bii,biii,ci,cii): 200 μm, (ciii): 50 μm.

In order to generate 3D vessels as experimentally tractable models with controlled geometries, techniques other than self-organization are necessary. Microphysiological systems (MPS) have been reported in which vascular cells are cultured in a large-diameter, patterned 3D lumen inside a microfluidic chip.^11^ These MPS are also referred to as “vessel-on-chip” (VoC) systems. Many different engineering techniques are being developed to produce more complex MPS. Most often, VoCs are fabricated by patterning an extracellular matrix with a small-diameter needle.^12,13^ This method yields reproducible lumens using a straightforward methodology but scaling up of experiments is labor intensive and removing the template without disturbing the structure is challenging. An alternative method is viscous finger patterning (VFP). VFP is a microfluidic technique that exploits the difference in viscosity between two fluids to generate a scaffold that can be used for cell culture.^14–16^ When a less viscous fluid displaces a viscous fluid in a confined channel, the less viscous fluid flows in a “fingerlike” shape through the middle of the channel; this is referred to as the Saffman-Taylor finger [Fig. 1(b,i–iii)].^17^ When the viscous fluid is a soluble hydrogel that can gelate, this results in a hollow structure resembling a lumen. The width of the Saffmann-Taylor finger is approximately half of the channel width and height assuming a sufficient difference in viscosity, no interface tension, and sufficient velocity.^18^ However, in practice, the dimensions achieved by several groups are approximately 60%–80% of the channel dimensions.^14,16^ A major benefit of VFP is that it can be easily scaled up as it only requires pipetting two fluids in a microfluidic channel. A downside of VFP to fabricate VoC systems is that the procedure to form the lumen is sensitive to many factors, such as extracellular matrix concentration and pH, timing, pressure, and inlet and outlet geometries. Fabrication of VoC systems can thus be challenging and inherently variable.

Besides the method of fabrication, the biological aspect also needs to be considered. Typically, *in vitro* models incorporate primary human cells.^6^ Although these cells are widely available from commercial sources or can be isolated directly from various human tissues, their supply is finite since they are not immortal. In addition, they exhibit donor-to-donor variability. This can negatively affect reproducibility and robustness of *in vitro* models. Human induced pluripotent stem cells (hiPSCs) can be used to generate vascular cells through differentiation with high reproducibility and robustness and therefore could improve cell quality and sustainability of VoC systems by allowing repeated return to the same (cryopreserved) singe cell source.^19–21^ hiPSCs are derived by reprograming somatic cells to a pluripotent state using four transcription factors: c-myc, Sox 2, Oct4, and Klf4.^22^ hiPSCs can be differentiated to all cells of the body including vascular cells if provided with the right growth factors. We have developed methods which support differentiation of ECs from hiPSCs (hiPSC-ECs) under defined culture conditions and have shown that these cells are functional in a multiplicity of assays *in vivo* and *in vitro.*^23,24^ hiPSCs can be derived from patients with specific disease genotypes or healthy control individuals with minimally invasive tissue collection.^25^ Furthermore, hiPSCs allow generation of cell types that are typically difficult to harvest from patients, like neurons from the brain or cardiomyocytes from the heart. Using advanced genetic modification, molecular indicators and markers can be introduced for live cell imaging and analysis.^26^ Using the same techniques, isogenic healthy control hiPSC lines can be derived by genetic repair of the disease-causing mutation, minimizing differences and allowing effects of the mutation of interest to be studied independent of genetic background.

Here, we describe a robust and reproducible method to generate VoC systems using an optimized VFP protocol for dynamic flow experiments. We use hiPSC-ECs, rather than those isolated from primary tissues or blood from donors, to allow the inclusion of genetically marked cells into the device so that they can be precisely tracked. The system we describe represents an important advance in the fabrication of robust, low-cost VoC systems that will enable future dynamic studies in disease modeling and drug development.

## RESULTS

To optimize the VFP-technique, straight flow channels with fixed dimensions of 500 *μ*m × 500 *μ*m *×* 1 cm [w *×* h *×* l, Fig. 1(a,i)] were designed to facilitate lumen formation. Master-moulds were fabricated with SU-8 photolithography to produce flow channels that were highly similar. Polydimethylsiloxane (PDMS) casting of the master-moulds was used to fabricate chips by conventional soft lithography. Analysis of the PDMS cast showed that, on average, channels had a width of 496 ± 3.7 *μ*m, a height of 527 ± 1.2 *μ*m, and an aspect ratio (AR) of 1.06 (data not shown).

First, lumens were patterned as described by Bischel *et al.* with a minor modification [protocol 1: passive pumping (PP), Fig. S1].^14^ This method relies on medium flow driven by the differential surface tension of a small droplet placed on the inlet and a large droplet on the outlet of the microfluidic channel. When the droplet on the outlet is sufficiently large, the force generated by passive pumping can be calculated using the following Eq. (2):^27^

$$\Delta P=\frac{2\gamma }{r},$$ (2)

where P: pressure (Pa), γ: surface tension (N/m), and r: radius curvature of the droplet (m).

**FIG. 2.**
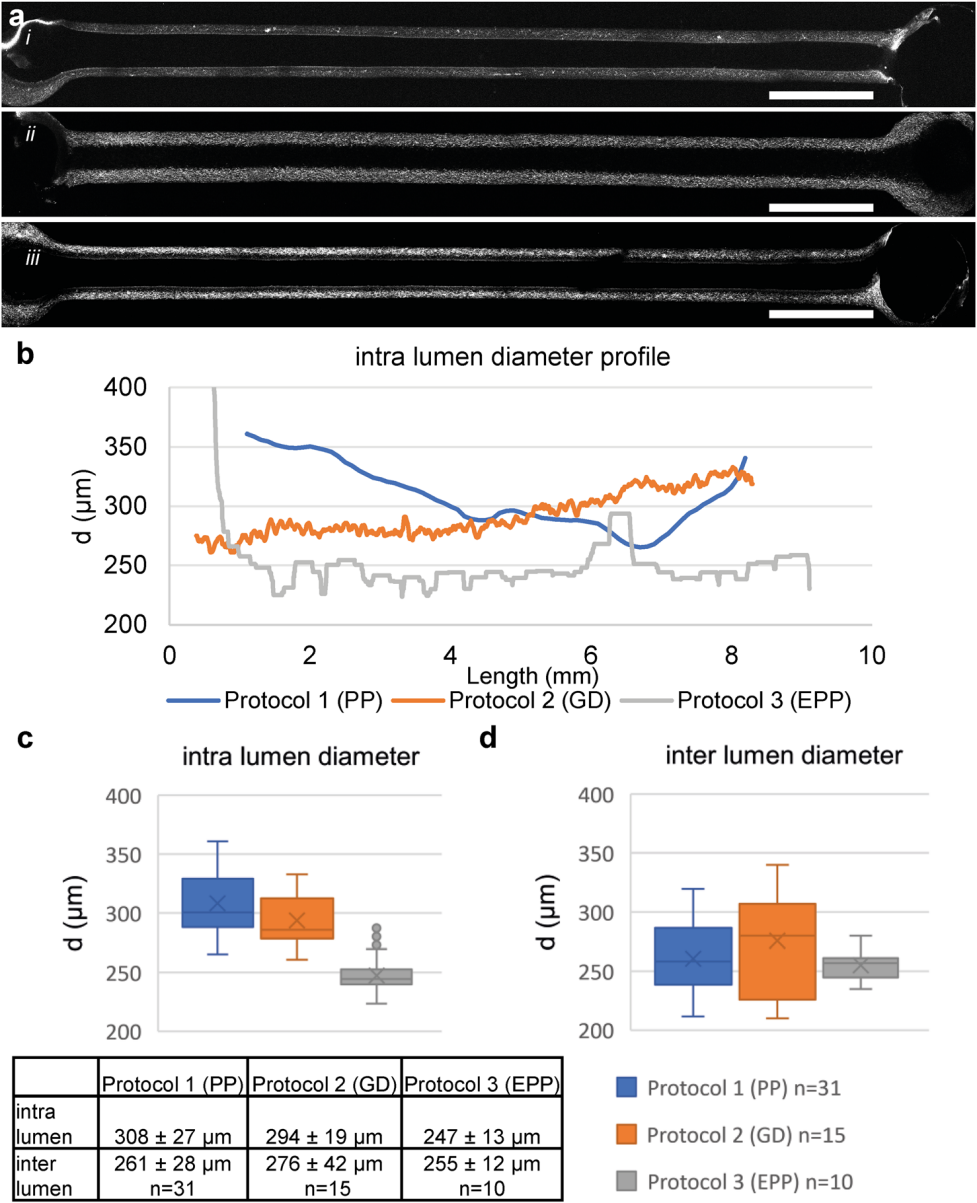
Comparison of different VFP protocols. (a) 2-Photon image of the middle slice of a representative lumen patterned with (i) passive pumping, (ii) gravity driven, (iii) extended passive pumping. (b) Intradiameter analyses of the representative lumen, the lumen patterned with PP (blue) shows a significantly gradually narrowing of the lumen, lumens patterned with GD (orange) show a straight lumen in the first part of the lumen followed by a gradual widening of the lumen. Lumens patterned with EPP (gray) show a uniform diameter of the lumen over the complete length, with a small oscillating trend. (c) Boxplot of the intradiameter of the represented lumen shows the large range and standard deviation in the PP patterned lumen. (d) Boxplot of interdiameter analysis of patterned lumen based on center 1.5 mm; PP (n = 31), GD (n = 15), EPP (n = 10). Boxplot displaying Q2-Q3- whiskers display Q1 and Q4, Dots: outliers, x: mean value. The Levene's test shows nonequal homogeneity of variance (p = 0.007); one-way ANOVA analysis shows no significant difference in means (p = 0.654). Scale bars: 1000 *μ*m.

This shows that a smaller initial droplet (i.e., higher curvature) generates more force. This curvature depends on the dimensions of the inlet, volume of the applied droplet, and hydrophobicity of the surface. Patterned collagen I was imaged using two-photon second harmonic generation [2P-SHG, Figs. 1(c) and 1(d)] and the diameter was analyzed. The diameter was defined as the widest part of the reconstructed flow area [Fig. 1(c,ii)]. In our hands, the protocol had a success rate (defined as generation of a perfusable lumen with the diameter <400 *μ*m) of 90%–100% depending on the operator. When comparing the lumen diameter in the middle section of the flow channel, we observed that the average lumen diameter was 261 ± 28 *μ*m, which is similar to that reported by others previously with comparable flow channel dimensions (cf. 256 ± 21 *μ*m).^15^ However, the analysis showed a significant entry effect so that these lumens gradually decreased in diameter over the length of the channel [Figs. 2(a,i) and 2(b), blue line]. Diameter analysis showed a high interlumen variation, with diameters ranging from 220 to 320 *μ*m [Figs. 2(d) and S3]. We further observed additional variation when this protocol was performed by different operators. Interestingly, we observed that the lumen diameter was typically the smallest at around 5–7 mm from the inlet [Fig. 2(b)].

We next patterned lumens by applying a modified protocol described by Herland *et al.*;^16^ which relies on hydrostatic pressure on the chip inlets to drive lumen formation. As reported previously and from our experience, air-bubbles were the major cause of failure with this protocol (data not shown). We therefore modified the protocol by introducing an additional step that included inserting an empty pipette tip prior to injecting the collagen [protocol 2: gravity driven (GD), Fig. S1(b)]. This allowed addition of phosphate-buffered saline (PBS) to the top of the collagen without introducing air bubbles. Lumens were imaged with 2p-SHG and the diameters measured [Figs. 2(a,ii), 2(b), orange line, and S4]. Interestingly, the lumens patterned following this adaption showed a uniform diameter at the beginning of the channel, with gradual widening of the lumen at the end [Figs. 2(a,ii) and 2(b), orange line]. The GD protocol had a high success rate (defined as a perfusable lumen with a diameter <400 *μ*m) and an improved intralumen diameter profile [Fig. 2(c)]; however, with the parameters used, this protocol was sensitive to small deviations in the volume and the height resulting in a relatively high interlumen variation [Fig. 2(d)]. We also noticed that the GD protocol failed with some batches of collagen possibly due to batch-to-batch differences. To successfully initiate patterning with these collagen batches, more pressure was required (i.e., more driver fluid) which resulted in a widening of the lumen like that reported by Herland *et al.*

In order to generate uniform lumens with more pressure and less volume, we combined the protocols for passive pumping and extension of the pathway. To consistently extend the pathway, pipette tips were cut using a customized mold that resulted in uniform end lengths of 7 mm [protocol 3: extended passive pumping (EPP), Fig. S1(a)]. Lumens were imaged with 2P-SHG and the diameter measured [Figs. 2(a,iii), 2(b), gray line, and S4]. Diameters typically showed a small oscillating trend around the average diameter across the length of the channel. All lumens (n = 10) had similar diameters with an average width of 255 ± 12 *μ*m [Figs. 2(d) and S4]. Levene's statistical test showed nonequal variance (p = 0.007), confirming smaller variance in the diameter of lumens patterned using the EPP protocol (protocol 3) than the lumens patterned using the PP or GD protocols (protocol 1 and protocol 2, respectively). As expected, one-way analysis of variance (AVOVA) analysis indicated no differences between the means (p = 0.654). In higher throughput experiments, the overall success was 88/96 (92%) of all attempts, defined as a perfusable lumen with diameters <400 *μ*m. The reason for failure was introduction of air bubbles that prevented patterning. To further optimize this protocol, we tested different path lengths to reduce the total collagen volume. By first cutting a pipette tip at 7 mm and then shortening this small tip to 2–3 mm, we kept the inlet diameter similar while reducing the total length. These shortened tips were inserted such that their total height was, respectively, 3 mm, 5 mm, and 7 mm from the coverslip. With the 3- and 5-mm extension, we observed an entry effect with a sudden decrease in the diameter followed by a consistent diameter, similar to what was observed in the PP protocol. With the 7 mm extension, this effect was not observed or was less distinct (Fig. S5).

**FIG. 3.**
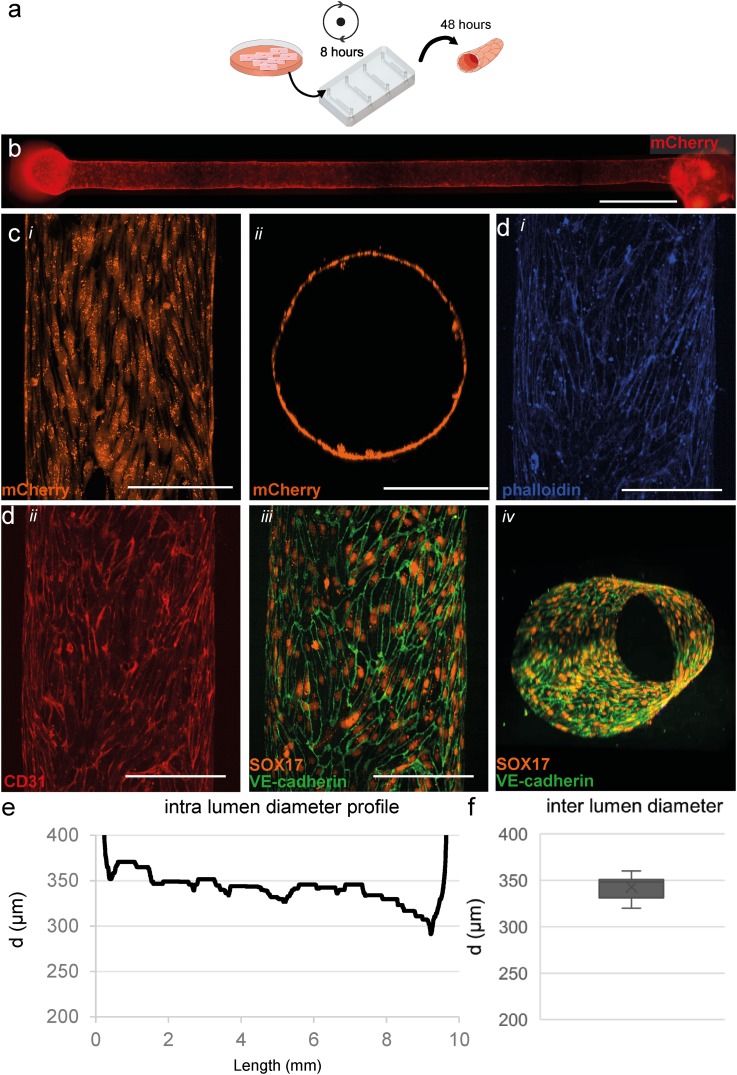
Three-dimensional cell culture of hiPSC-ECs. (a) Schematic overview of cell seeding procedure and culture in microfluidic devices. hiPSC-ECs were seeded and cultured for 48 h in static conditions. (b) Widefield image shows an even and consistent mCherry signal demonstrating uniform coverage of hiPSC-ECs along the whole lumen in a collagen scaffold. (c), (i) Top-down view of live cell confocal image, (ii) XY-reconstruction of the live cell confocal microscopy confirms complete coverage around the perimeter of the lumen. (d) Top-down reconstruction of the lumen visualized using the following markers. (i) F-actin (phalloidin, visualized in blue), (ii) CD31 (visualized in red), and (iii) VE-Cadherin (visualized in green) at the periphery of the hiPSC-ECs costained with SOX17 (visualized in orange) localized at the nuclei of endothelial cells showing alignment with the longitudinal axis of the lumen, and (iv) 3D reconstruction of the engineered vessel showing VE-cadherin and SOX17 around the complete periphery of the lumen and a more detailed reconstruction is presented in video S1. (e) Analyses of the full-length channel show a uniform diameter with small tapering near the outlet. (f) Diameter analysis of cellularised lumens (n = 8), on average 343 ± 12 *μ*m. Scale bars, (b): 1000 *μ*m, (c) and (d): 200 *μ*m.

In parallel, next to the patterning protocol, we also optimized cell seeding into the scaffolds. hiPSCs were genetically engineered to express the fluorescent protein mCherry ubiquitously and differentiated to ECs as described previously.^23,24,28^ This allowed live cell imaging in 3D in real time. We tested various seeding densities (data not shown) and found that 1 × 10^7^ cells/ml was optimal for complete- and uniform channel coverage and high cell viability. The microfluidic devices were slowly rotated for 8 h to allow even cell attachment around the complete lumen [Fig. 3(a)]. After 48 h, live cells were imaged using an EVOS fluorescence microscope [Fig. 3(b)]. Even distribution of fluorescence from the mCherry expressing ECs was observed across the entire length of each channel. Further analyses using confocal fluorescence microscopy confirmed even coverage around the perimeter of the lumen [Fig. 3(c,i,ii)]. Analysis of the vessel diameter (n = 8) showed an average width of 343 ± 12 *μ*m [Fig. 3(f)]. The diameter profile was essentially uniform but was on average significantly wider than that of bare collagen lumens. VoCs were fixed and immunostained for the endothelial-specific markers CD31, SOX17 and VE-cadherin [Fig. 3(d)]. VE-cadherin was observed at the cell-cell junctions of the ECs, demonstrating uniform EC interaction and monolayer formation [Fig. 3(d,iii)].

**FIG. 4.**
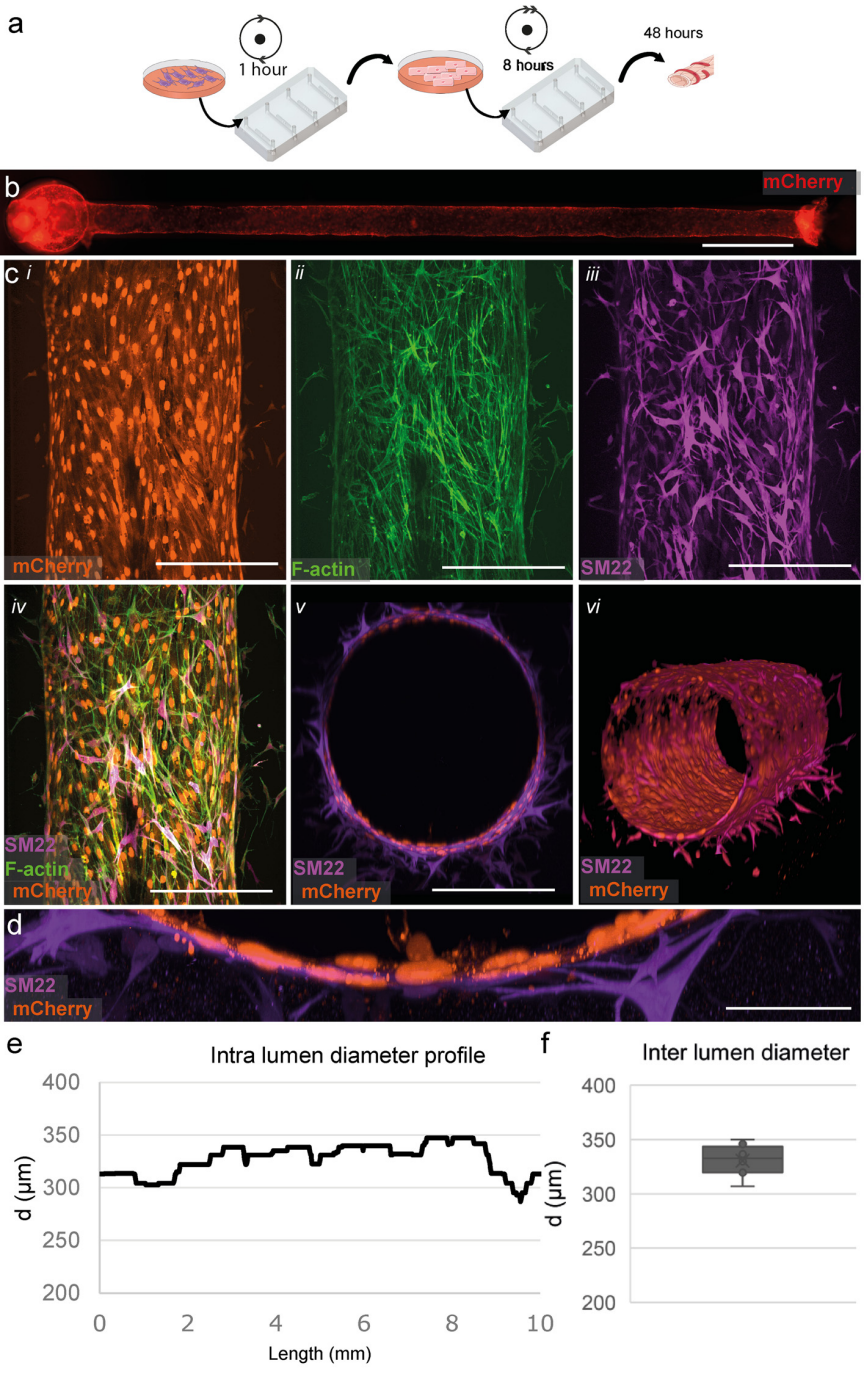
Three-dimensional coculture of hiPSC-ECs and HBVPs. (a) Schematic overview of the protocol for cell seeding and culture in microfluidic devices. (b) Widefield image shows an even and consistent mCherry signal demonstrating uniform coverage of hiPSC-ECs along the whole lumen in collagen gel, similar to that of the monoculture. (c) Top-down view of immunofluorescent staining showing close interaction of EC and pericytes. (i) mCherry expressing ECs (visualized in orange). (ii) f-actin (visualized in green). (iii) SM22 staining HBVPs (visualized in magenta) and (iv) merged image of EC- and mural cell-specific markers. (v) XZ-view demonstrating lumen lined with mCherry labeled ECs (orange) surrounded by HBVPs (magenta). (vi) 3D reconstruction of the vascular tube, a complete reconstruction is presented in video S2. (d) High magnification of cross section, demonstrating close interaction between the inner EC-layer and surrounding HBVP supporting cells. (e) Diameter analyses of a full-length channel show a uniform channel similar to monoculture. (f) Diameter analysis of the cellularized channel, on average 331 ± 13 *μ*m (n = 8). Scale bars, (b) 1000 *μ*m, (c) 200 *μ*m, and (d) 50 *μ*m.

To further recapitulate the architecture of *in vivo* vasculature, we generated 3D vessels with supporting mural cells (Fig. 4). First, we seeded primary human brain vascular pericytes (HBVPs) into the lumen scaffold and rotated for 1 h [Fig. 4(a)]. Next, hiPSC-ECs were seeded as described before and cultured for 48 h. Uniform coverage by hiPSC-ECs was observed across the entire length of the scaffold similar to the monoculture [Fig. 4(b)]. HBVPs were also found evenly distributed across the microfluidic channel. This was evident in immunofluorescence images taken using spinning disk confocal microscopy which showed a uniform endothelial cell layer with adjacent mural cells, as visualized by mCherry (hiPSC-ECs), F-actin (hiPSC-ECs and HBVPs), and SM22 (mural cell-specific marker to label HBVPs) [Fig. 4(c,i–vi), supplementary video]. Notably, close interaction was observed between the HBVPs and hiPSC-ECs [Fig. 4(d)]. Importantly, measurement of the diameter of these vessels in the cocultures was comparable to that in the monocultures 331 ± 13 *μ*m [n = 8, nonsignificant p value = 0.18, Fig. 4(f)]. Typically, the diameter profile of the cocultures shows a similar uniform expansion.

**FIG. 5.**
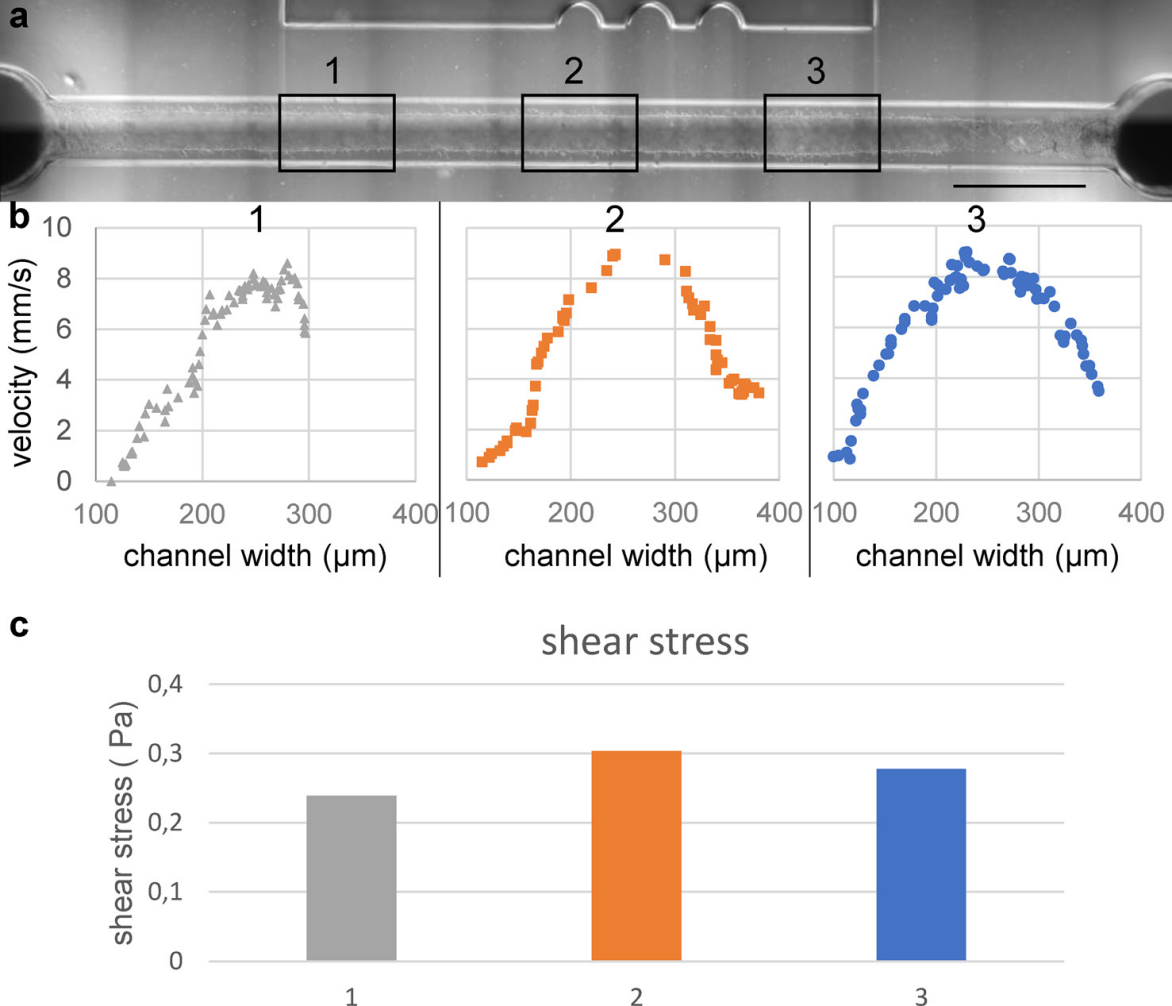
Analysis of the flow profile. (a) Widefield image of the perfused lumen. Boxes are showing the predetermined regions of interest (ROI) at 2.5 mm, 5 mm, and 7.5 mm from the inlet. The average diameter of ROI 1: 320 *μ*m, ROI 2: 290 *μ*m, and ROI 3: 300 *μ*m. A full impression of the perfused lumen is presented in video S3. (b) Velocity profile reconstructed of 30 frames per ROI showing maximum velocity in the center of the lumen. Some interaction of the beads with the cell wall can be observed. Using maximum velocity and assuming laminar flow the volumetric flow rate is determined to be, respectively, 19 *μ*m/min, 18 *μ*m/min, and 18 *μ*m/min. (c) Calculated shear stress per ROI using the determined flow rates and measure diameters. ROI 1: 0.24 Pa, ROI 2: 0.30 Pa, and ROI 0.28 Pa. Scale bar: 1000 μm.

To further explore the applicability of the technology in functional studies, such as shear-stress analysis and modeling endothelial-leukocyte interaction under flow, we determined the flow profile across the entire length of the channel of 3D vessels generated using the optimized EPP protocol. The flow profile was determined by the assessment of the velocity of the fluorescent beads that were perfused at a specific flow rate (20 *μ*l/min). Distances traveled by individual beads were manually measured at the specific segments of the channel to reconstitute the flow profile [Figs. 5(a) and 5(b)]. Shear stress was calculated from the maximum velocity and diameter at the specific segments of the channel [Fig. 5(c), N = 1]. The cultured lumen was segmented into three Regions of Interest (ROI 1–3) and the average diameter was determined: ROI (ROI 1: 320 *μ*m, ROI 2: 290 *μ*m, ROI 3: 300 *μ*m). Flow profile analyses showed that on average the flow rates are comparable with each other and the set flow rate [ROI 1: 18.8 ± 1.3 *μ*l/min, ROI 2: 17.8 ± 1.5 *μ*l/min, ROI 3: 18.0 ± 0.6 *μ*l/min (cf. set flow rate: 20 *μ*l/min)]. The corresponding shear stress was calculated using Eq. (1). This analysis showed that, on average, shear stress was 0.27 Pa with a maximum of 13% deviation within the ROIs thus validating the channels as being capable of producing equal sheer stresses along their length.

## DISCUSSION

To generate robust *in vitro* 3D vascular models, it is essential to balance bioengineering with cell biology; cell survival and functionality must be compatible with scaffold design. An important feature for high-utility technology is that it yields technical replicates regardless of operators. Currently, 3D vessels are fabricated by encapsulating and removing a small-diameter needle or PDMS rods in an extracellular matrix.^12,29–32^ Although this method is straightforward and reproducible, it is laborious and difficult to scale up. To address this, we investigated the VFP described by Beebe *et al.* and showed it to be easily implementable and scalable but we observed gradual narrowing of the patterned lumen along its length. This change in the lumen shape is a challenge in the context of dynamic studies since the ECs are subject to significantly different shear stresses when exposed to flow (up to threefold in a single channel). Shear stress plays a pivotal role in cell fate and behavior and intralumen variation would reduce the uniformity of results a priori in a single channel and require elaborate downstream analyses to interpret results. Diameter variation should therefore be kept to a minimum.^10,33^

In this study, we showed that by modifying the existing protocol and by extending the entry length, we could rapidly generate reproducible 3D vascular structures with reduced variance without increasing difficulties in the technical preparation or requiring specialized equipment. The method proved to be scalable and typically 24 channels could be patterned by one person within 10 min. The protocol is low cost and can be easily implemented in a nonspecialist biology lab. We further showed that these 3D vascular structures could support the culture of either hiPSC-ECs or cocultures of hiPSC-ECs and primary mural cells (HBVPs).

To generate circular lumens, we designed flow channels with a square cross section. However, since manufacturing variability can be expected, dimensions were verified by analyzing the cross section of a PDMS cast. This showed that the resulting flow channel has a width approximately as designed (500 cf. 496 *μ*m). However, the height appeared to be consistently 529 *μ*m, giving an aspect ratio (AR) of 1.06. It was therefore expected that the patterned lumens would also have an AR of 1.06.

In confocal microscopy, it is essential to use an immersion medium that exactly matches the refractive index of the sample to minimize differences in z-reconstruction.^34^ The refraction index of a collagen I hydrogel has been reported to be 1.336, close to that of water (1.333).^35^ However, depending on the structure, concentration, and hydration this value variable ranges from 1.3 to 1.5.^36,37^

To investigate how this effect influences our diameter analyses, we imaged one lumen with three different immersion media. We observed that although the height was significantly affected by this refraction index mismatch, the width was not (Fig. S6). Therefore, the diameter can be estimated by measuring the maximum width of a reconstructed lumen regardless of immersion medium or dry objectives. Dry objectives have major benefits in that they have typically longer working distance ranges and are able to switch to multiple fields during image acquisition, improving the throughput of confocal analyses.

As reported by Bischel *et al.*, the diameter of the patterned lumen depends on the viscosity of the collagen, the velocity of patterning, and the dimensions of the microfluidic channel. The viscosity of collagen is highly dependent on its concentration.^38^ Therefore, mixing a homogenous collagen solution is crucial for reproducibility between experiments. The stock solution of highly concentrated rat-tail collagen is extremely viscous and adhesive to standard pipette-tips and deviations were observed between different mixing batches. To optimize reproducibility between experiments, we found it best that the quantity of collagen be verified by weight and then diluted accordingly. Furthermore, we observed during the throughput optimization step that when many lumens were patterned from a single mixing batch, those last showed inconsistent lumen shapes. For this reason, we rigorously mixed the collagen, prepared it in small batches, and used it immediately for optimal reproducibility.

We observed that most lumens generated using protocol 1 tended to have a minimum diameter of 5–7 mm from the inlet of the channel. By contrast, we observed a stable diameter at the beginning of the lumens patterned with protocol 2 and propose that extension of the pathway prior to the microfluidic channel could improve the lumen diameter profile.

To extend the pathway, we inserted 7 mm cut pipette tips into the outlet prior patterning for three reasons. First, this prevents the narrowing of the patterned lumen near the inlet. By removing both pipette tips while twisting, the inlet and outlets are collagen-free allowing easy connection to a microfluidic flow system. Second, the shape of the driving droplet is not dependent on the hydrophobicity of the PDMS. The hydrophobicity of the microfluidic device changes after plasma treatment and surface functionalization and posttreatment, in a time-dependent manner and this introduces an extra variable.^39^ By using an untreated, defined material, this hydrophobicity is constant and results in a constant driving force. Finally, patterning is less dependent on manufacturing errors like damaged or bent inlets by punching or casting variability. To cut these tips with a consistent length, we made a custom cutting guide. Using 3 mm and 5 mm extensions, we observed a distinct entry effect with a constriction at the inlet of the channel. Using the 7 mm extension, this entry effect was reduced or vague. It is important to use only straight cut tips of consistent length as an entry effect can be observed when tips are too short. To optimize the total length, we tested different tip lengths using the same diameter. Further extension could remove this entry effect completely; however, more collagen is required. Therefore, 7 mm is designated as optimum.

The patterned lumens are elastic and are significantly deformed by cell seeding during injection; however, we observed that this deformation is uniform and reproducible when similar pressures were applied during cell seeding. It is important to point out that introduction of air bubbles severely deform the patterned lumens and must be avoided. This stable diameter ensured more uniform physiological conditions for cells seeded in the lumen, as evidenced by particle image velocimetry, allowing more accurate dynamic experiments to be performed. ∼0.3 Pa is a physiologically low shear stress; however, with our current system, higher velocities could not be measured as the individual particles were not detected in two successive frames.

## CONCLUSION AND FUTURE OUTLOOK

Lumens patterned using our protocol with standardized parameters resulted in high reproducibility in the fabrication of VoC systems without specialized equipment. It also proved to be rapidly scalable and applicable to vascular structures consisting of hiPSC-derived vascular cells. Furthermore, these vascular models were perfusable with minimal variation in shear stress. This will eventually allow isogenic cells to be cultured as 3D vessels *in vitro* that recapitulate *in vivo* architecture. Combination of vascular and inflammatory cell types differentiated from hiPSCs may help to determine which cellular component of the vessel wall is affected by (genetic) disease and cause the disease phenotype. This platform was demonstrated to be fully compatible with various microscopes in conventional biological workflow. While we used straight channels here, the protocol is easily adapted to curved channels with similar results (Fig. S7). This can be relevant for some pathologies that result in different fluid mechanical conditions, such as atherosclerotic plaques in blood vessels, and could be modeled with this adaptable protocol.

## METHODS

### Generation of mCherry expressing hiPSC line

NCRM-1 hiPSC line was obtained from RUDCR Infinite Biologics at Rutgers University [originally generated by NIH Center for Regenerative Medicine (NIH CRM)], additional information available in public database: https://hpscreg.eu/cell-line/CRMi003-A. Ethics approval is not required. The NCRM-1 line is a male control reference line obtained from CD34+ cord blood cells and is reprogramed with an episomal plasmid. The cell line was modified in-house with a mCherry expression cassette under the human cytomegalovirus (CMV) early enhancer/chicken β actin (CAG) promoter using a previously established protocol.^40^

### Differentiation hiPSC-ECs

hiPSC-ECs were differentiated using a previously reported protocol with minor modifications.^23,28^ Briefly, hiPSCs were maintained in TeSR™-E8™ medium on vitronectin-coated 6-well plates and seeded at day-1. Twenty-four hours after seeding E8 medium was replaced with B(P)EL medium supplemented with 8 *μ*M CHIR. On day 3, the medium was replaced with B(P)EL medium supplemented with 50 ng/ml VEGF (R&D systems) and 10 *μ*M SB431542 (Tocris Bioscience) and refreshed on days 6–9. hiPSC-ECs were isolated on day 10 using CD31-Dynabeads™ (Invitrogen) as previously described.^28^ hiPSC-ECs from cryo-preserved batches were used in all further experiments.

### Thawing and expanding of hiPSC-ECs

hiPSC-EC cells were thawed, resuspended in complete Endothelial cell serum-free medium (EC-SFM, Gibco, cat. no. 11111‐044), and plated on a 0.1% gelatine-coated culture flask in complete EC-SFM, as previously described.^28^ Cells were used for experiments when nearly confluent by visual inspection, typically on day 4. Cells were harvested using TrypLE™ according to the manufacturer's instructions. All hiPSC-ECs were used at passage #2.

### Fabrication of microfluidic devices

Master-moulds for the microfluidic devices were designed in SolidWorks (Dassault Systèmes SolidWorks Corp.) and fabricated in a cleanroom by SU-8 photolithography at the University of Twente. Dimensions of flow channels were 1 cm × 500 *μ*m × 500 *μ*m [l × w × h, Fig. 1(a,i)]. To fabricate the microfluidic devices, a polydimethylsiloxane (PDMS, Sylgard 184, Dow Corning) base agent was mixed 10:1 with a curing-agent and poured onto the master-mould to yield a PDMS slab with a thickness of approximately 5 mm. This thickness created a more stable platform for pipetting without deforming the channels by manual handling. The PDMS was degassed for one hour at room temperature under vacuum and cured at 75 °C for 180 min. After curing, the PDMS was cooled to room temperature and carefully peeled off. Inlet holes were punched with Uni-Core punch (1.2 mm, GE Healthcare) and the microfluidic chip was cut to fit the cover glass. Round cover glasses (#1.5, ø 34 mm Thermo scientific) were spin-coated with 100 *μ*l PDMS (5 s 1000 RPM, 30 s 4000 RPM, PDMS prepared as described above). The spin-coated cover glass and the prepared microfluidic devices were air plasma treated (2 min, 50 mA at 20 Pa) and contact bonded. To functionalize PDMS to covalently bind collagen I and support the scaffold, a 2-step process was carried out immediately after contact bonding. 5% (v/v) (3-Aminopropyl)-triethoxysilane (APTES, Sigma-Aldrich) was prepared fresh in distilled water (Gibco^®^), injected into the channels, and incubated for 30 min at RT. Channels were thoroughly rinsed with absolute ethanol and dried using a nitrogen-gas flow. Channels were subsequently injected with 10% (v/v) Glutaraldehyde (Sigma-Aldrich, in dH_2_O) and incubated at RT for 30 min. Channels were thoroughly rinsed with absolute ethanol, dried with a nitrogen gas flow, and baked at 75 °C for at least 5 h to evaporate any residual ethanol and glutaraldehyde. It is essential to remove any residual glutaraldehyde and ethanol as this will affect cell viability.

### Preparation of collagen I solution

The preparation of collagen I is an important step for reproducible lumens. High concentration rat tail collagen I (Corning) was prepared to a final concentration of 5 mg/ml on ice. Briefly, a stock solution was prepared using M199 medium 10× (Gibco), distilled water (Gibco), and 1 M sodium hydroxide according to the manufacturer's instructions. The appropriate amount of collagen I was pipetted in an empty vial using a positive displacement-pipette and weighed to verify quantity. The proper amount of stock solution was added to the collagen and carefully mixed. pH was adjusted with sodium hydroxide by adding until the color of the mixture changed from yellow to pink indicating a pH value of approximately 7.4. The pH of collagen I is crucial for the mechanical properties; therefore care must be taken to perform this step as reproducibly as possible.^41^ The collagen I mixture was always thoroughly mixed and spun down to remove air bubbles and kept on ice. Mixed collagen was used within 10 min.

### Pattering of lumens using viscous finger patterning

A schematic representation of all methods is given in Fig. S1. The protocol as published by Bischel *et al.* was performed with a minor adaption as presented in Fig. S1 protocol 1. To be able to connect the microfluidic devices to a microfluidic pump, a large outlet port was not possible. For this reason, we left a pipette tip filled with collagen behind to reduce the surface tension. The protocol published by Herland *et al.* was modified to improve the success-rate by inserting an empty pipette tip before injecting the collagen (Fig. S1 protocol 2: GD). This allows applying the PBS to induce the needed hydrostatic pressure without a manual error.

Our protocol (Fig. S1 protocol 3 EPP) is as follows. A standard P10 pipette tip (Greiner Bio-One #741015) was cut at approximately 7 mm from the tip using a custom fabricated cutting guide and inserted into the outlet of the microfluidic channel. 10 *μ*l of collagen I mixture was injected via the inlet of the channel with a P10 pipette tip until the meniscus of the collagen I mixture reached the outlet of the microfluidic channel. The pipette tip was carefully and smoothly ejected from the pipette, keeping the meniscus at the outlet and leaving the pipette tip and the remaining collagen in the inlet. To achieve this, when attaching the pipette tip to the pipette, it is important to press only lightly to ensure a smooth release. Immediately, 3.5 *μ*l of PBS was carefully pipetted on top of the collagen in the small tip to initiate patterning. Patterning typically takes 15 s and can be confirmed by disappearance of the applied droplet and a rise in the collagen level in the opposite tip. Alternatively, PBS can be supplemented with contrasting food dye for visual confirmation of patterning. However, this affects surface tension and requires an extra washing step before cell seeding as the compounds might influence cells or (fluorescent) microscopy. Immediately after patterning lumens were incubated for 30 min at 37 °C in a humid incubator. After 30 min, EGM-2 medium (Lonza) was pipetted in the pipette tip and channels were incubated overnight at 37 °C to allow the pH to set. Prior to cell seeding, pipette tips were removed in a smooth twisting motion. To prevent the formation of air bubbles and deformation of the patterned collagen, the cut pipette tip was first removed and a new pipette tip was inserted. The medium was then added to this pipette tip and the intact pipette tip was removed. A new pipette tip was then carefully inserted. These pipette-tips aid injecting cells into the channels and act as medium reservoirs.

### Three-dimensional cell-culture

Primary human brain vascular pericytes (HBVP, ScienCell) were obtained and cultured in pericyte medium (ScienCell). Cells were harvested using TrypLE, and resuspended at a concentration of 3.3 × 10^6^ cells/ml. 10 *μ*l of cell suspension was carefully injected using gel loading pipette-tips. Care was given not to introduce any air-bubbles that might deform the collagen scaffold. This was achieved by generating a small droplet in the pipette-tip before inserting. The microfluidic device was put on a rotator (0.4 RPM, channel longitudinal axis in line with rotating axis) to allow even distribution of cells for 1 h at 37 °C. hiPSC-ECs were harvested as described above and resuspended at a concentration of 1 × 10^7^ cells/ml. 5 *μ*l of the cell suspension was carefully injected using gel loading pipette-tips. The microfluidic device was put on a rotator (0.4 RPM, channel longitudinal axis in line with rotating axis) and slowly rotated for 8 h at 37 °C to ensure complete coverage of the lumen. For monocultures, only ECs were seeded. Microfluidic devices were placed in a 6-well culture plate and incubated under static conditions at 37 °C. dH_2_O was added between wells to prevent evaporation. Culture Medium (EGM-2+ pen/strep) was supplemented with VEGF (50 ng/ml) and refreshed every 24 h.

### Two-photon second harmonic generation

Unstained Collagen I was imaged using two-photon second harmonic generation (2P-SHG) with a Leica SP5 confocal microscope and a multiphoton laser tuned at 810 nm. Emitted light was detected with a BG38 bandpass filter (CWL 470) before the detector.

### Flow profile analysis

Microfluidic channels were placed on a microscope and connected to a 1 ml syringe pump (Cole Palmer) using 45° 20 g blunt needles. The culture medium was supplemented with 5 *μ*m fluorescent beads and the flow rate was set to 20 *μ*l/min. The flow was allowed to settle for 1 min after which 30 frames per location were recorded at a predetermined location (2.5 mm, 5 mm, 7.5 mm of the channel) at a frame rate of 30 frames per second. All frames were combined per location to reconstitute the flow profile and the maximum velocity was determined. The average diameter of the location was determined using a widefield image and the local shear stress was calculated using Eq. (1) using the calculated values assuming the viscosity of 37 °C medium reported as 0.0008^8^.

### Live cell imaging

Wide-field microscopy was performed using an EVOS FL auto 2 microscope with a 4× (NA 0.4) air objective. Live cell confocal microscopy was performed using a Leica SP5 inverted microscope using mCherry excitation/emission settings and a 20× (NA 0.75) water immersion objective.

### Immunofluorescence staining

Cells were fixed by injecting 4% paraformaldehyde (PFA) solution into the channels and incubated for 15 min at RT. Cells were subsequently permeabilised using 0.1% (v/v) Triton-X 100 in PBS(−) for 10 min at RT. Channels were blocked with 1% bovine serum albumin (BSA) in PBS(−) (w/w) for 30 min at RT. Primary antibodies were diluted (1:200) and injected into the channels and incubated overnight at 4 °C. Samples were rinsed three times with PBS(−). Secondary antibodies were diluted (1:300) in 1% BSA and injected into the channels. Channels were incubated at RT for 1 h and washed three times with PBS(−) for 10 min. 4′,6-Diamidino-2-phenylindole (DAPI) was used to stain cell nuclei. After washing steps, pipette-tips were filled with PBS(−) to prevent drying out and were stored in the dark at 4 °C until imaging.

### Fluorescence imaging

Immuno-stained cells were imaged using a Leica SP8 microscope equipped with a Dragonfly^®^ spinning disk (Andor) using 20× (NA 0.75) water objective or 63× oil (NA 1.4).

### Image handling

Images generated with the Leica SP5 were handled using Fiji ImageJ software. Images generated with the Leica SP8 spinning disk were handled with Imaris software (Bitplane, Oxford Instruments). For a top-down view, a full Z-stack was halved. A maximum projection of the halved stack was made to visualize the top monolayer.

### Diameter analyses

In Fiji ImageJ, Z-stacks were resliced with 10 *μ*m spacing to XZ-view to visualize the formed lumen. For 2p-SHG, the stack was smoothed to reduce gaps and the average intensity and standard deviation inside the lumen were measured. A threshold value was calculated with the following equation:

Threshold=Average mean+3×Stdv. (3)

The ImageJ magic wand-tool was programmed to trace the inner lining, and an ellipse was fitted. From the measurement, the width of the object was retrieved. Measurements under 100 *μ*m and above 400 *μ*m were excluded and replaced by an interpolated value. Full-length channels were plotted against the length. For inter lumen analyses, 1.5 mm around the center of the channel was measured and the average diameter was taken as the diameter of that channel. Cell covered lumens were manually measured using an XZ-reconstruction and measured outside the mCherry signal. Results are shown as average diameter ± standard deviation.

### Statistical analysis

Statistical analyses were performed with the IBM SPSS 25 software package. A Kolmogorov-Smirnoff test was performed to test for normal or uniform distribution. A test of homogeneity of variances (Levene's test) was performed to compare variances of the analyzed diameters. A one-way ANOVA test was performed to compare the means.

## SUPPLEMENTARY MATERIAL

See the supplementary material for a detailed overview of all viscous finger patterning protocols performed, 2P-SHG cross sections of all lumens analyzed, representative diameter analyses of the pathway length optimization and the XZ reconstruction, and analyses of a lumen imaged using different immersion media. Supplementary videos of the 3d reconstruction of the monoculture and cocultures and an example of the flow profile are also available.
